# The ability of damselfish to distinguish between dangerous and harmless sea snakes

**DOI:** 10.1038/s41598-020-58258-2

**Published:** 2020-01-28

**Authors:** Claire Goiran, Richard Shine

**Affiliations:** 1 LabEx Corail & ISEA, Université de la Nouvelle-Calédonie, BP R4, 98851 Nouméa cedex, New Caledonia; 2 Department of Biological Sciences, Macquarie University, NSW 2109 Sydney, Australia; 3 School of Life and Environmental Sciences, University of Sydney, NSW 2006 Sydney, Australia

**Keywords:** Behavioural ecology, Ecosystem ecology, Evolutionary ecology

## Abstract

In defence of their nests or territories, damselfish (Pomacentridae) attack even large and potentially dangerous intruders. The Indo-Pacific region contains many species of sea snakes, some of which eat damselfish whereas others do not. Can the fishes identify which sea snake taxa pose a threat? We recorded responses of damselfishes to natural encounters with five species of snakes in two shallow bays near Noumea, New Caledonia. Attacks by fishes were performed mostly by demersal territorial species of damselfish, and were non-random with respect to the species, size, sex and colouration of the snakes involved. The most common target of attack was *Emydocephalus annulatus*, a specialist egg-eater that poses no danger to adult fishes. Individuals of a generalist predator (*Aipysurus duboisii*) that were melanic (and thus resembled *E. annulatus* in colour) attracted more attacks than did paler individuals. Larger faster-swimming snake species (*Aipysurus laevis*, *Laticauda saintgironsi*) were watched but not attacked, or were actively avoided (*Hydrophis major*), even though only one of these species (*A. laevis*) eats pomacentrids. Attacks were more common towards female snakes rather than males, likely reflecting slower swimming speeds in females. In summary, damselfishes distinguish between sea snake species using cues such as size, colour and behaviour, but the fishes sometimes make mistakes.

## Introduction

Individuals of many species are under significant risk of predation and hence, experience intense selection to optimize their responses to potential predators^1,2^. One major challenge is to match the response to the magnitude of threat; for example, the best tactic may be to flee if the predator poses a major risk, but to attack if the predator poses little risk to the individual involved but a substantial risk to its offspring^3^. Multiple types of predators may often be present within the same area, increasing the challenge of correct predator identification^4,5^. Simply treating all potential predators as dangerous is the safest option, but may entail high costs (e.g., of disrupted feeding, interrupted courtship, less effective parental care) from unnecessarily cautious behaviour^6,7^.

In keeping with these ideas, an extensive literature documents sophisticated responses by potential prey to potential predators. For example, the alarm calls of birds can encode information not only about predator species^8^, but also predator behaviour^8,9^ and body size^10^. Likewise, fishes can discriminate between hungry and satiated predators^11^ and salamanders can distinguish the faeces of snakes fed on salamanders versus on alternative prey^12,13^. Lizards can distinguish between high-threat and low-threat predators^5,7,14,15^. Antipredator responses can be innate and/or learned^8,16^ and can change rapidly depending on local conditions^17,18^.

Predation is a major threat to survival for many reef-dwelling fishes^19^, stimulating the widespread evolution of abilities to detect and evade approaching predators^20–22^. In Indo-Pacific coral reef ecosystems, sea snakes from two main lineages (hydrophiine and laticaudine elapids) are abundant and diverse (around 80 species^23^) and prey primarily upon fishes^24,25^. Although sea snakes encompass a diversity of body shapes and colours, many taxa are grey to brown with transverse darker bars^25,26^. Perhaps reflecting their recent origin^27^, many “true” (hydrophiine) sea snakes are “notoriously difficult” for most biologists to identify to species^28,29^. Despite those morphological similarities, however, sea snakes exhibit diverse diets. Some taxa feed mostly or entirely on a single type of fish (e.g., *Hydrophis stokesii* eats only scorpionfish^24^) whereas others take a broad range of taxa (e.g., *Aipysurus laevis* has been reported to consume fish from 12 families^24,30^). Often, even closely related (and thus, morphologically similar) species diverge in diets: for example, *Aipysurus eydouxii* specializes on fish-eggs whereas *A. duboisii* is a generalist predator^24^.

Sea snakes are abundant and speciose in many parts of the Indo-Pacific, with several species often occurring within the same small area^25,26^. Even more abundant and diverse are fishes of the family Pomacentridae (damselfish)^31,32^. In New Caledonian waters, 113 species of Pomacentridae have been recorded^33^. We might expect damselfish to have evolved to recognize which sea snakes are dangerous for themselves or for their clutch and which are not, for the following reasons. First, damselfish lay their eggs on the substrate where they are preyed upon by many other animals, including snakes. Parental defence of the nest is therefore important^34^. Second, damselfish have good visual acuity; they are brightly coloured, and use visual cues to identify fish species when initiating responses to intruders^35,36^. Third, damselfish are capable of learning to discriminate between different types of predators^6,36–39^, even through social cues^40,41^. They can associate different cues^42^, generalize predator recognition to congeneric species^39,43^, and assess predation risk^44–48^. To evaluate the ability of damselfishes to discriminate between sea snakes that consume or do not consume these fish (or their eggs), we gathered standardized data on snake-fish interactions in the course of our fieldwork in two small bays near Noumea, in New Caledonia.

## Materials and Methods

### Study site

Baie des Citrons and Anse Vata are small (approx. 1 km wide) bays beside Noumea, New Caledonia (22°18′10″S, 166°26′08″E). The substrate is dominated by live coral, coral rubble, and sand^49^. Water depth at high tide in our study site ranges up to 3 m, but extensive areas remain < 1 m deep even at high tide. Water temperature during the observations averaged 24.04 °C (SD = 1.78 °C) and water depth averaged 1.80 m (SD = 0.71 m). Observations were spread evenly over the tidal cycle (low 35%, medium 25%, high 40%).

### Study species

**Figure 1:**
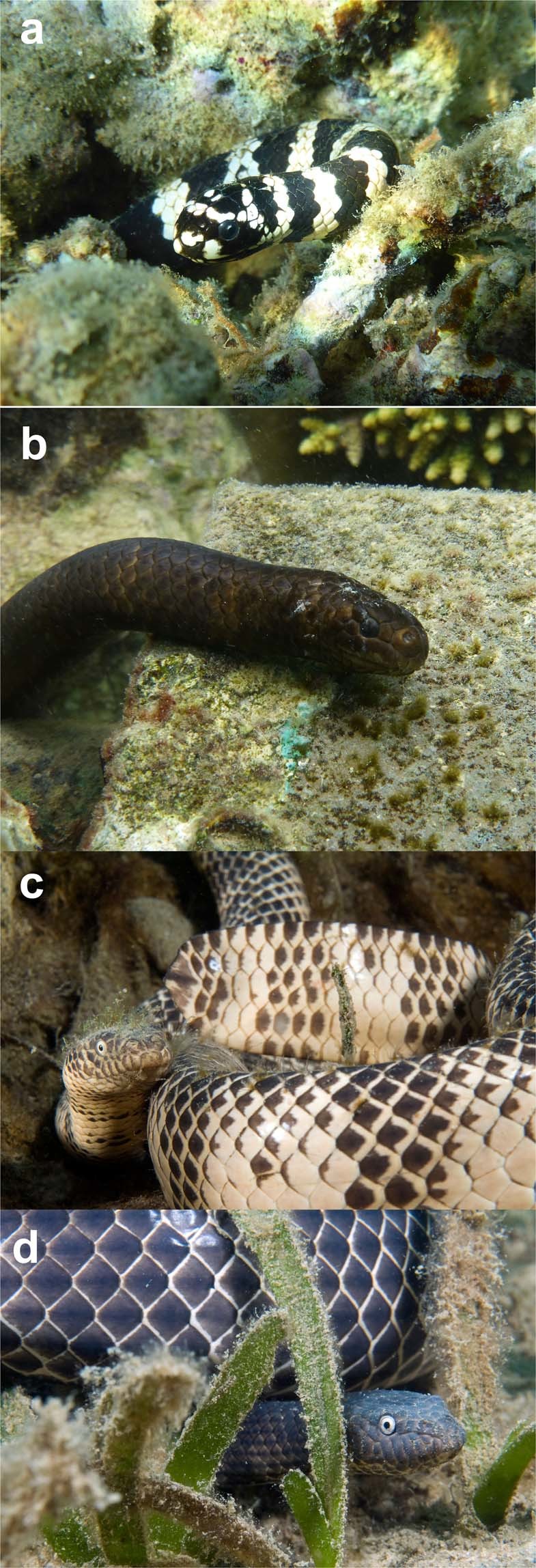
Photographs of colour morphs of two sea snake species found in the study area. Both *Emydocephalus annulatus* (**a,b**) and *Aipysurus duboisii* (**c,d**) occur in both light and melanic colour phases. Photographs by Claire Goiran (**a,b**) and Yves Gillet (**c,d**).

We obtained data on 113 natural interactions between fishes and snakes (see Supplementary Materials 1 for video footage of interactions) during fieldwork between February 2012 and September 2016. Most observations were made as a snorkeler swam slowly along behind a moving snake. Observations of snake-fish interactions were made throughout the year (January, N = 20 records, February, N = 21; March, N = 15; April, N = 3; June, N = 2; August, N = 19; September, N = 12; October, N = 7; November, N = 1; December, N = 13). Of the snakes, 30 observations were of the Reef Shallows Sea Snake *Aipysurus duboisii*, 3 of the Olive Sea Snake *A. laevis*, 22 of the Turtle-Headed Sea Snake *Emydocephalus annulatus*, 30 of the Greater Sea Snake *Hydrophis major*, and 28 of the Yellow-Lipped Sea Krait *Laticauda saintgironsi* (see Table 1 and Fig. 1 for photographs). The two *Aipysurus* species are generalist piscivores, and thus pose a potential risk to adult damselfish^24^. The other snake species have specialized diets that do not include adult damselfish: *Emydocephalus annulatus* eats only the eggs of fishes, *Hydrophis major* feeds mainly or exclusively on catfish, and *Laticauda saintgironsi* specializes on eels^24,29,34^.

**Table 1:** Species of sea snakes and fishes observed in interactions in the Baie des Citrons and Anse Vata, Noumea. Table shows average body lengths of snakes (from Ineich and Laboute 2002), and maximum sizes of fishes (the latter from FishBase. Available at: https://fishbase.org/).

Common name	Scientific name	Body length (mm)
**Snakes**		
Reef Shallows Sea Snake	*Aipysurus duboisii*	700–800
Olive Sea Snake	*Aipysurus laevis*	1100–1500
Turtle-Headed Sea Snake	*Emydocephalus annulatus*	500–800
Greater Sea Snake	*Hydrophis major*	1100–1500
Sea Krait	*Laticauda saintgironsi*	800–1100
**Fishes**		
Scissortail Sergeant	*Abudefduf sexfasciatus*	190
Orbicular Damselfish	*Amblyglyphidodon orbicularis*	83
Big-lip Damselfish	*Cheiloprion labiatus*	60
Twospot Damselfish	*Chrysiptera biocellata*	125
Banded Humbug	*Dascyllus aruanus*	100
Coral Damselfish	*Neopomacentrus nemurus*	80
Obscure Damselfish	*Pomacentrus adelus*	85
Ambon Damselfish	*Pomacentrus amboinensis*	90
Goldenbrow Damselfish	*Pomacentrus aurifrons*	62
Yellowtail Damselfish	*Pomacentrus chrysurus*	90
Lemon Damselfish	*Pomacentrus moluccensis*	90
Nagasaki Damselfish	*Pomacentrus nagasakiensis*	110
Dusky Gregory	*Stegastes nigricans*	140
Bluntsnout Gregory	*Stegastes punctatus*	130

The fish species involved in interactions with snakes comprised 38 cases with *Pomacentrus adelus*, 33 with *P. moluccensis*, 15 with *Cheiloprion labiatus*, 9 with *Stegastes* sp., 5 with *Amblyglyphidodon orbicularis*, 4 with *P. chrysurus*, 2 with *P. nagasakiensis*, and single cases with *Abudefduf sexfasciatus*, *Chrysiptera biocellata*, *Dascyllus aruanus*, *Neopomacentrus nemurus*, *P. amboinensis*, and *P. aurifrons*. An additional single case involved either *Stegastes nigricans* or *S. punctatus* (the two species are difficult to discriminate when underwater). These fish species span a wide range of sizes, colours and ecologies (Table 1). Some of the interactions doubtless involved the same individual snakes and individual fishes but we could not unambiguously identify them and thus, treat each fish-snake encounter as an independent observation.

### Data collection methods

In the course of other behavioural-ecology studies^34,50,51^, one of us (CG) snorkelled over fringing reefs, taking notes, photographs and videos documenting the behaviour of sea snakes and fishes. These studies were conducted during daylight hours only (0753 h to 1439 h). CG also swam along belt transects in the same area, performing visual fish censuses (5 transects 30 m long and 2 m wide, between fixed points, 3 visual censuses of damselfish, gobies and blennies on each transect per year, in May or June, at high tide, between 0830 and 1030 h) over the period May 2012 to May 2018. We used the cumulative number of each fish taxon within the total survey effort as a measure of relative abundance, to compare to the frequencies with which individual fish species were recorded to attack snakes.

We recorded tide (high-low-medium) and water temperature at the beginning of each survey session, and recorded water depth at each observation. Each snake was identified to species and classified into one of three size classes (small, medium, large relative to other members of the same species). We determined sex of adult snakes based on tail shape^25,52^. We also estimated sizes of fishes involved and took notes on aspects such as colour of the snake (for species with different colour morphs).

We defined an interaction between fishes and snakes as any case in which a fish modified its behaviour as a result of the approach of a snake. We classed those behaviours as “flee”, “watch” and “attack”. We scored an interaction as “attack” if even a single fish out of the group bit or hit the snake. We restricted the current study to pomacentrids because these are abundant at our study site, and they comprised the majority of fishes seen to interact with snakes. In a previous study, we examined nest-defence tactic of gobies and blennies (as well as pomacentrids) towards Turtle-Headed Sea Snakes attempting to consume demersal eggs of those fish species^34^. The present study looks at responses of damselfish to additional snake taxa. We never saw gobies or blennies interacting with any snake species other than *E. annulatus*.

As well as looking at overall patterns, we examined fish responses to *Aipysurus duboisii* more closely, because this was the only snake species for which we recorded substantial intraspecific variation in fish responses (i.e., bite vs. not bite the snake). That variation allowed us to investigate factors associated with the responses of fish to snakes.

### Statistical analysis

Using JMP Pro 13 (SAS Institute, Cary, NC), we checked normality and variance homogeneity of all continuous variables prior to analysis. We used contingency-table analysis to compare the relative abundances of different genera of fish (based on our overall surveys) with the numbers recorded to attack snakes. We used ANOVA with “species of snake” as the factor to explore variation in the mean sizes and numbers of fishes interacting with different species of snake. To compare conditions where fish attacked versus did not attack, we used that variable as the factor in an ANOVA with either water depth or water temperature as the dependent variable. We used nominal logistic regression to see if the suite of fish genera involved differed among snake species. To evaluate determinants of fish responses, we used nominal logistic regression with whether or not the fish bit the snake as the dependent variable, and snake species and size class (or sex) as the factors. To compare fish responses to tidal phase, we used tide phase (high-low-medium) as the factor, and fish response (attack snake vs. not) as the dependent variable in a nominal logistic regression. We included interaction terms between factors as appropriate, but deleted them if they were non-significant (*p* > 0.05) and recalculated the analyses with main effects only. Where analyses yielded significant results, we used Tukey-Kramer posthoc tests to identify the location of significant differences.

## Results

### Species of fishes interacting with snakes

**Figure 2:**
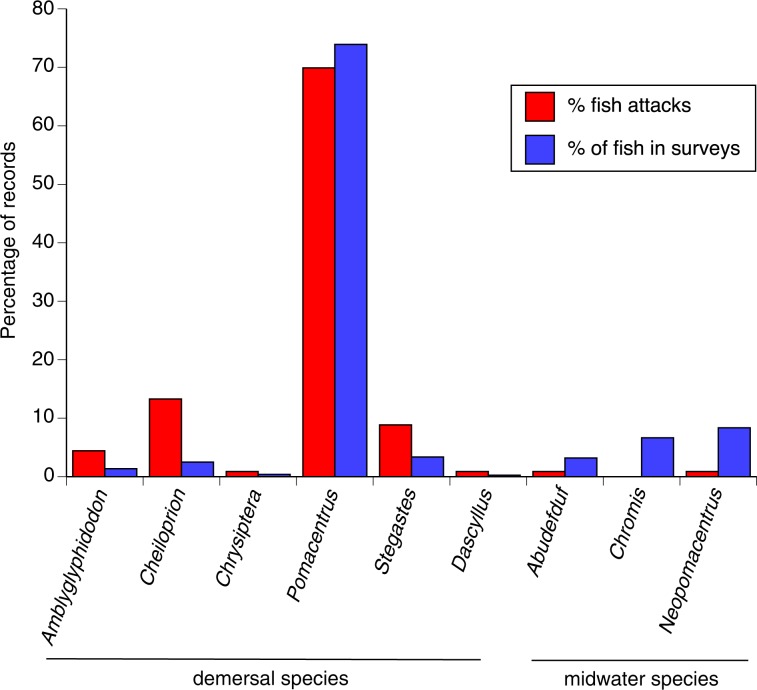
The abundance of damselfish of each genus (based on overall counts during surveys) compared to the number of interactions with snakes recorded for damselfish of each genus. In both cases, numbers are shown as a proportion of total records (*N* = 7327 for surveys, *N* = 113 for interactions with snakes). The damselfish genera are divided into those that defend demersal territories, and midwater species that defend against intruders only while they have active nests.

We recorded 7327 fish during our habitat surveys, encompassing nine genera of damselfish (Table 1). The 113 attacks on snakes were primarily by demersal territorial genera (*Cheiloprion, Dascyllus, Amblyglyphidodon, Pomacentrus, Stegastes*) rather than by mid-water taxa that only exhibit active defence when they are nesting (*Abudefduf, Neopomacentrus, Chromis*: see Fig. 2). Contingency-table analysis confirmed that the number of attacks relative to fish abundance differed among genera (χ^2^ = 68.08, df = 8, *p* < 0.0001) and was higher for demersal (territorial) than for midwater (nest-defending) genera (χ^2^ = 30.98, df = 1, *p* < 0.0001).

### Species and numbers of fishes per interaction

The genera of fish recorded to interact with snakes did not differ significantly among the five snake taxa that we studied (nominal logistic χ^2^ = 26.66, df = 28, *p* = 0.5). However, the mean size of fish interacting with *Hydrophis major* (6.33 cm) was greater than that for all other species except *Aipysurus laevis* (mean 5.00 cm; *F*_1,4_ = 4.12, *p* < 0.004; posthoc tests show HM > LS = AD = EA; all species means except HM were 5.0 to 5.4 cm). The mean number of fishes involved per interaction also differed among snake species (*F*_1,4_ = 3.44, *p* < 0.015), ranging from 1.8 for *E. annulatus* to 21.0 for *A. laevis* (means from 4.1 to 6.9 in the other three taxa; posthoc tests show AL = LS = AD; LS = AD = HM = EA; AL > HM = EA).

### Effects of snake traits on probability of attack by fishes

**Figure 3:**
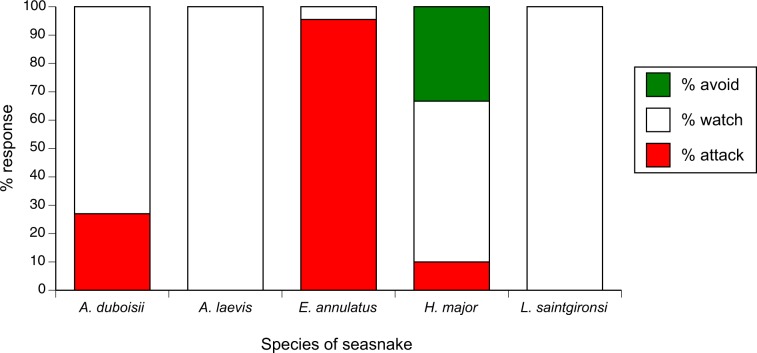
Responses by pomacentrid fishes to encounters with sea snakes of five species (*Aipysurus duboisii*, *Aipysurus laevis*, *Emydocephalus annulatus*, *Hydrophis major*, *Laticauda saintgironsi*).

Fish were more likely to bite some snake species than others (χ^2^ = 54.63, df = 4, *p* < 0.0001) and were less likely to bite small snakes than larger ones (χ^2^ = 6.79, df = 2, *p* < 0.035; interaction term NS so deleted). Inspection of the data show that fish rarely or never bit three species of snakes (*A. laevis*, *H. major*, *L. saintgironsi*), bit *A. duboisii* in about one-quarter of the encounters, and almost always bit *E. annulatus* (Fig. 3).

**Figure 4:**
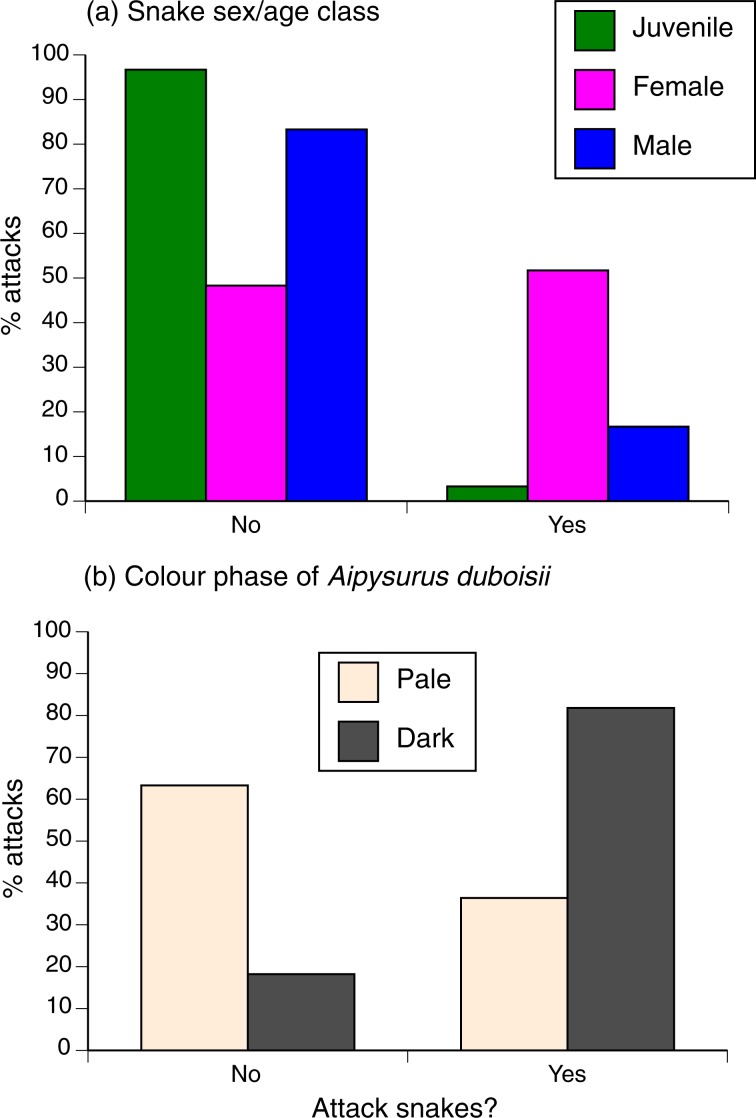
Effects of a snake’s sex/age class (upper panel) and colour (lower panel) on the probability that it was attacked by damselfish (“no” = snake was not attacked; “yes” = snake was attacked). The upper panel is based on data from all snake species, whereas the lower panel is based only on data from the colour-polymorphic species *Aipysurus duboisii* (see Fig. 1 for photographs of those colour phases).

The only species that sometimes attracted bites and sometimes did not (and thus, in which we can further explore determinants of fish attacks) is *Aipysurus duboisii*. This species shows substantial variation in colouration among individuals within the local population, with melanic individuals resembling melanic *E. annulatus*^53^ whereas other individuals are pale (see Fig. 1c,d). Logistic regression showed that dark-coloured specimens were more likely to be bitten than were pale-coloured individuals (χ^2^ = 4.68, df = 1, *p* < 0.035) and that females were more likely to be bitten than males (8 of 15 females bitten, vs. 0 of 15 males; χ^2^ = 9.17, df = 1, *p* < 0.003; interaction NS so deleted; see Fig. 4). Bites were directed to the head in 22 of 32 cases where we could accurately score bite locations (69%), with no significant difference between snake species in this respect (χ^2^ = 1.81, df = 2, *p* = 0.41).

### Environmental influences on responses by fishes

Interactions that involved fishes biting snakes occurred in deeper water (mean of 2.13 m) than did interactions in which no bites were recorded (1.68 m; ANOVA, *F*_1,106_ = 9.97, *p* < 0.003). This effect was not due to different species of snakes being encountered at different depths, because the difference in water depth remained significant after “snake species” was added into the analysis (effect of snake species *F*_4,106_ = 147.33, *p* < 0.0001; effect of water depth *F*_1,106_ = 9.60, *p* < 0.003; interaction NS so deleted). In the sole species of snake that attracted variable responses (*Aipysurus duboisii*), the depth effect remains significant (*F*_1,28_ = 18.41, *p* < 0.0002; mean depth for bites 2.19 m, for no bites 1.32 m).

One potential influence on this water-depth pattern is that snake size may covary with water depth^49^; larger snakes are found in deeper water (in the overall dataset, snake size class vs. water depth: *F*_2,109_ = 5.37, *p* < 0.006). The same pattern was evident within each of the snake species for which we had data on a range of size classes (*Emydocephalus annulatus F*_2,19_ = 10.99, *p* < 0.004; *Hydrophis major F*_2,27_ = 4.09, *p* < 0.03; *Laticauda saintgironsi F*_2,25_ = 29.07, *p* < 0.0001).

### Effects of snake traits on probability of avoidance by fishes

Broadly, the factors that resulted in fishes fleeing from snakes were the opposite of those that stimulated attacks. That is, fishes were less likely to flee if the snake was in deeper water, and if it was a female rather than a male or juvenile. The only species of snake that elicited rapid retreat by fishes was *H. major*, with fish fleeing in 10 of 30 interactions (comparison among species χ^2^ = 29.39, df = 4, *p* < 0.0001; see Fig. 3). Within fish that encountered *H. major*, flight was elicited more often by juvenile snakes (7 of 11 cases) and males (3 of 5 cases) rather than by females (0 of 14 cases; effect of sex-age class χ^2^ = 17.04, df = 2, *p* < 0.0002).

### Determinants of responses by snakes

When bitten by fishes, 8 of 8 *A. duboisii* fled, as did 18 of 21 *E. annulatus*. No snakes of other species were bitten, and none fled. Rapid retreat was more likely if the snake was bitten than if it was not bitten (in *A. duboisii*, χ^2^ = 24.79, df = 1, *p* < 0.0001). A nominal logistic regression with “snake fled or not” as the dependent variable revealed significant effects of snake species (χ^2^ = 24.80, df = 4, *p* < 0.0001), snake size class (larger snakes fled more often; χ^2^ = 6.05, df = 2, *p* < 0.05), snake sex (females were more likely to flee: χ^2^ = 10.54, df = 1, *p* < 0.0015) and whether or not the snake had been bitten by a fish (χ^2^ = 10.72, df = 1, *p* < 0.0015).

## Discussion

Despite their small body size relative to the snakes that entered their territories (typically, a 60 mm fish vs. 1-m-long snake), damselfish are very pugnacious; some species even attack human divers^54^. One of us (CG) was bitten several times by *Stegastes* sp. while collecting data for this study. Hence, it is not surprising that these small fishes often attacked snakes that entered the fish’s territory. The same species of fishes were involved in attacks on all types of snakes. Comparisons with overall abundances (from broad surveys) indicate that the fishes attacking snakes are a non-random subset of the damselfish taxa in the area. Specifically, most attacks on snakes were by damselfish species that defend demersal territories against all comers. Other damselfish taxa, most often seen above the substrate and pugnacious only when defending their nests, accounted for the remaining cases of attacks on snakes.

Anecdotal observations of damselfish biting sea snakes have been recorded by several authors, but always against *Emydocephalus*^26,55,56^. We recorded attacks against additional species, and these attacks broadly can be divided into two main types of interactions. For one species, the small and slow-moving egg-predator *E. annulatus*, fish almost invariably attacked the snake and often repulsed it (as described in detail for gobies and blennies as well as damselfish^34^). In contrast, fishes almost never attacked three larger, faster-moving species (*A. laevis*, *H. major*, *L. saintgironsi*). The only exception to this dichotomy was *A. duboisii*, which is intermediate both in size and in swimming speeds, and was attacked on about 25% of our observation periods. Intriguingly, this species (a generalist piscivore, and thus a potential danger to adult damselfish) exhibits substantial variation in colouration – and individuals that were dark (and hence, resembled the harmless *E. annulatus*) were attacked more often than paler individuals (that resembled the larger hydrophiine species).

Interactions between fishes and *E. annulatus* were also distinctive in involving a single fish, whereas most interactions with all of the other snake taxa involved groups of fishes. Numbers may confer safety in this situation, rendering it less likely that an individual fish will be seized by the predator^57^. Broadly, our data on *E. annulatus* support and extend our earlier analysis in the same site, focusing on gobiid and blenniid responses to egg-searching snakes^34^. Our data on responses to the other snake species suggest that these potentially dangerous animals are treated quite differently than is the harmless *E. annulatus*, albeit with significant attacks to one of those piscivorous taxa (*A. duboisii*). The concentration of attacks on dark-coloured individuals, and on slow-moving females rather than fast-moving males, suggests that these are cases of mistaken identification by the fishes.

At our study site, some *E. annulatus* are strongly black-and-white banded rather than melanic^53^. Thus, banded individuals of *E. annulatus* resemble sea kraits (*L. saintgironsi*), a species that was never attacked (Fig. 1a and Fig. 3). Why then did fishes direct attacks to banded as well as melanic *E. annulatus*? If fishes use colour as a criterion for recognizing snake species (as suggested by attacks to melanic *A. duboisii*), we might have expected them to misidentify banded *E. annulatus* as another more dangerous species. In practice, however, *E. annulatus* are also distinctive in their heavyset morphology and very slow movements; and these additional criteria may enable fish to recognize this species regardless of its colour.

In keeping with an influence of non-chromatic cues, the responses by fishes were also affected by snake body size. Juvenile snakes were rarely attacked. One possible reason is that these snakes were too small to pose a threat, because sea snakes are gape-limited predators^58^. However, all of the juvenile snakes that we saw were large enough to swallow most of the fishes that were seen interacting with snakes; and certainly were large enough to swallow eggs of fishes. Thus, we doubt that juvenile snakes were tolerated because they were too small to pose a threat. Instead, the lack of attacks may reflect a tendency for juvenile snakes to be encountered in shallow water, where attacks would be more risky for fishes (because of limited space to evade retaliation by the snake, plus the danger from aerial predators when a fish is focused on the snake). For many other systems, larger predators likely pose more risk and hence attract more intense responses by potential prey (e.g., ground squirrels assessing risk from rattlesnakes^59^).

Intriguingly, the probability of attack was also affected by the sex of the snake involved in the encounter. Sexual dimorphism and dichromatism are minor in the snake species involved, suggesting that the differential responses of fishes to male versus female snakes were driven by differences in behaviour rather than morphology. At our study site, most of the female snakes we see are foraging, a behaviour that entails slow movement near the reef structure with frequent tongue-flicking as the snake searches for chemical cues of prey presence. In contrast, males (especially of the four larger species) are more often encountered while they are searching for mates. Of seven males unequivocally scored as “mate-searching” (5 *A. duboisii*, 2 *H. major*), none were attacked by fishes. Mate-searching behaviour involves rapid movement. (e.g., in *E. annulatus*, swimming speeds of mate-searching males are more than 3 times greater than for foraging snakes^60^). In at least one of the species we studied (*A. laevis*), males are rarely seen in shallow reef sites (such as our study area) except during the mating season^61^. Higher swimming speeds may give the fish less time to react before the snake leaves the fish’s territory; and also, may show that the snake is not foraging, and hence is not a direct threat.

In summary, pomacentrid fishes are astute but not infallible at identifying snakes. The response of fishes to snakes may reflect general tactics (reactions to any animal that intrudes into their territory) rather than selection for optimal solutions to the risk posed by snakes. Thus, for example, large animals moving quickly, especially in shallow water, may best be treated as potentially dangerous and hence either watched or avoided. In contrast, a slow-moving snake in deeper water may allow time for fish to assess the degree of threat posed by the snake (to the fish as well as to its eggs) and to react accordingly. Although the potential costs of attacking a piscivorous predator are high, the actual risk may be slight because snakes rarely seize prey in open water. Most sea snakes catch their prey inside crevices in the coral or adjacent soft-bottom substrates^25,60,62^, although some species can capture free-swimming fishes (e.g., *Hydrophis platura*^63^). We never saw a snake strike at a fish that was attacking it; instead, the fish were ignored or avoided.

Future research could usefully explore the generality of our results. For example, how are the responses of fishes affected by environmental factors (such as turbidity of the water) and what role (if any) do chemical cues play? Chemical cues may be important in predator-prey interactions in many aquatic habitats^64,65^, but remain poorly understood^66^ and may be affected by anthropogenic disturbance (e.g., ocean acidification^67^). The species of snakes and fishes that we studied are widespread, providing an opportunity to repeat our analyses in different sites. Lastly, the rapid development of robotic models should make it possible for investigators to manipulate aspects such as the size, shape, colour and swimming speeds of snake-shaped objects, and quantify fish responses in an experimental framework.

## Supplementary information

Electronic supplementary material.

ESM 1.

ESM 2.

ESM 3.

ESM 4.

ESM 5.

ESM 6.

ESM 7.

ESM 8.
