# Supplementary material for: The ability of damselfish to distinguish between dangerous and harmless sea snakes
Source: Sci Rep. 2020 Jan 28;10:1377. doi: 10.1038/s41598-020-58258-2 (PMC6987208; doi:10.1038/s41598-020-58258-2)
Supplement: Supplementary file 1 — Electronic supplementary material. [file 41598_2020_58258_MOESM1_ESM.docx]

**Electronic supplementary material**

**The ability of damselfish to distinguish between dangerous and harmless sea snakes**

**Claire Goiran and Richard Shine**

**ESM Video 1** Lemon Damselfish *Pomacentrus moluccensis*attacking a melanic Reef Shallows Sea Snake *Aipysurus duboisii.* Video by Claire Goiran. cgoiran-7ef3ed601537db2b8090639c1e582f0e-565553

**ESM Video 2** Lemon Damselfish *Pomacentrus moluccensis*attacking a melanic Reef Shallows Sea Snake *Aipysurus duboisii.* Video by Claire Goiran. cgoiran-9055332f561fe9bcccb877f381b54346-473746

**ESM Video 3** Obscure damselfish *Pomacentrus adelus* attacking a melanic Reef Shallows Sea Snake *Aipysurus duboisii.* Video by Claire Goiran. cgoiran-cbd699e1957a0b74012e6db5dd18d41a-874401

**ESM Video 4** Banded Humbug *Dascyllus aruanus* attacking a melanic Turtle-Headed Sea Snake *Emydocephalus annulatus.* Video by Claire Goiran. cgoiran- 99942d630aaa0de10185b1ec13c6960c-620270

**ESM Video 5** Banded Humbug *Dascyllus aruanus* attacking a melanic Turtle-Headed Sea Snake *Emydocephalus annulatus.* Video by Claire Goiran. cgoiran- 07e69614145aeadecdea84abcf6bb8f-533456

**ESM Video 6** Banded Humbug *Dascyllus aruanus* attacking a melanic Turtle-Headed Sea Snake *Emydocephalus annulatus.* Video by Claire Goiran. cgoiran- be6ae1de4fb8eb47a309270311365008-666998

**ESM Video 7** Banded Humbug *Dascyllus aruanus* attacking a melanic Turtle-Headed Sea Snake *Emydocephalus annulatus.* Video by Claire Goiran. cgoiran- 9c9fd23cfdc1ed269a2ad1b23b13ad97-058193

**ESM Video 8** Obscure damselfish *Pomacentrus adelus* attacking a melanic Turtle-Headed Sea Snake *Emydocephalus annulatus.* Video by Claire Goiran. c cgoiran-2080a73a6bb467e34c997e0f0b930db5-659970
